# A population-based approach to integrated healthcare delivery: a scoping review of clinical care and public health collaboration

**DOI:** 10.1186/s12889-019-7002-z

**Published:** 2019-06-07

**Authors:** Mohammad Shahzad, Ross Upshur, Peter Donnelly, Aamir Bharmal, Xiaolin Wei, Patrick Feng, Adalsteinn D. Brown

**Affiliations:** 1 Dalla Lana School of Public Health, University of Toronto, Toronto, Ontario Canada; 2 Faculty of Medicine, University of Toronto, Toronto, Canada; 3 Division of Clinical Public Health, Dalla Lana School of Public Health, Toronto, Canada; 4 Department of Family and Community Medicine, University of Toronto, Toronto, Canada; 5 Bridgepoint Collaboratory, Lunenfeld-Tanenbaum Research Institute, Sinai Health System, Toronto, Canada; 6 Public Health Ontario, Toronto, Canada; 7 Fraser Health Authority, Surrey, British Columbia Canada; 8 Institute of Health Policy, Management and Evaluation, Dalla Lana School of Public Health, Toronto, Canada

**Keywords:** Population health, Primary care, Public health, Community health, Health equity

## Abstract

**Background:**

A population-based approach to healthcare goes beyond the traditional biomedical model and addresses the importance of cross-sectoral collaboration in promoting health of communities. By establishing partnerships across primary care (PC) and public health (PH) sectors in particular, healthcare organizations can address local health needs of populations and improve health outcomes. The purpose of this study was to map a series of interventions from the empirical literature that facilitate PC-PH collaboration and develop a resource for healthcare organizations to self-evaluate their clinical practices and identify opportunities for collaboration with PH.

**Methods:**

A scoping review was designed and studies from relevant peer-reviewed literature and reports between 1990 and 2017 were included if they met the following criteria: empirical study methodology (quantitative, qualitative, or mixed methods), based in US, Canada, Western Europe, Australia or New Zealand, describing an intervention involving PC-PH collaboration, and reporting on structures, processes, outcomes or markers of a PC-PH collaboration intervention.

**Results:**

Out of 2962 reviewed articles, 45 studies with interventions leading to collaboration were classified into the following four synergy groups developed by Lasker’s Committee on Medicine and Public Health: *Coordinating healthcare services* (*n* = 13); *Applying a population perspective to clinical practice* (*n* = 21); *Identifying and addressing community health problems* (*n* = 19), and *Strengthening health promotion and health protection* (*n* = 21). Furthermore, select empirical examples of interventions and their key features were highlighted to illustrate various approaches to implementing collaboration interventions in the field.

**Conclusions:**

The findings of our review can be utilized by a range of organizations in healthcare settings across the included countries. Furthermore, we developed a self-evaluation tool that can serve as a resource for clinical practices to identify opportunities for cross-sectoral collaboration and develop a range of interventions to address unmet health needs in communities; however, the generalizability of the findings depends on the evaluations conducted in individual studies in our review.

From a health equity perspective, our findings also highlight interventions from the empirical literature that address inequities in care by targeting underserved, high-risk populations groups. Further research is needed to develop outcome measures for successful collaboration and determine which interventions are sustainable in the long term.

**Electronic supplementary material:**

The online version of this article (10.1186/s12889-019-7002-z) contains supplementary material, which is available to authorized users.

## Background

The population health approach describes a shift in our healthcare system from a narrow model of acute care targeted at the individual patient, to one that focuses on the health and overall wellness of the broader population it serves [1]. In doing so, this approach highlights that clinical care, such as primary care, is only one of a wide range of ‘institutions’ that impact health [2]. Additionally, public health efforts, with a greater focus on health promotion and chronic disease prevention, can complement clinical care in order to provide populations with a comprehensive set of promotive, preventive and curative health services, thereby promoting overall population health [1].

Increasingly, healthcare systems around the world are facing persistent pressures that result in poor performance and growing inequities in care [3]. These include a rising burden of illness attributable to major chronic diseases, as well as increasing costs and complexity of healthcare delivery [4, 5]. To address these growing pressures and achieve a shift from our traditional biomedical model of healthcare to one that prioritizes wellness of populations [6], various authors have highlighted cross-sectoral collaboration as a key feature of the population health approach [7–10]. In particular, the US Institute of Medicine (IOM) [11] and others [12–15] have noted the role of collaboration between primary care (PC) and public health (PH) sectors in achieving lasting improvements in health outcomes. In their report on PC and PH integration, the IOM point out that pressing health needs within populations such as management of non-communicable diseases, maternal and child health, and cancer prevention fall within the scope of both PC and PH, yet these sectors are largely functioning independently of each other.

Importantly, various jurisdictions around the world have developed integration efforts involving PC and PH. Across Europe, for example, as part of the Health 2020 European Policy Framework, several countries are implementing collaborative models of healthcare and strengthening PH capacity in clinical settings [16]. Furthermore, with the introduction of Clinical Commissioning Groups (CCGs) in the UK in 2013, healthcare underwent a significant shift towards integrated, population-based delivery [17, 18] Additionally, in Canada, a number of provinces are developing initiatives in line with community-based priorities and population needs-based funding models to promote health of local communities [19, 20]. However, even in these jurisdictions, the attempts at collaboration are, at best, fragmented across the respective health systems [3].

There are, however, some concerns that merging clinical care and PH resources will lead to the “tyranny of the urgent” – the idea that demand for acute PC services will take precedence over PH needs for resources and investments that only show results in the long term [21]. Elsewhere, PH academics and practitioners have pointed out that combining their sector’s activities with PC and community care will limit the scope of PH, if due consideration is not given to different functions and expertise of all sectors involved [22]. For these reasons, we need to understand what such a transformation involving clinical care and PH would look like practically.

Previous reviews on PC and PH collaboration have highlighted different aspects of partnership while also advocating for greater interaction. The IOM report outlines core principles necessary for effective integration [11], Levesque et al. make note of overlapping functions and activities of both domains [23], and Martin-Misener et al. identify facilitators and barriers of PC-PH collaboration at systemic, organizational and interpersonal levels [24]. Furthermore, an Institute for Healthcare Improvement project on clinical-community linkages identifies high-level foundational steps involved in collaboration [25]. Significantly, they conclude that there are various conceptual models and principles in the literature, but these alone are not sufficient for healthcare practices seeking to design collaboration efforts for a defined geographic population. Similarly, these reviews have not found any significant efforts to produce practical tools for frontline clinicians, PC practices, and PH units to achieve collaboration.

We conducted a scoping review to identify specific interventions adopted by healthcare organizations internationally that resulted in collaboration between clinical care and PH. By analyzing empirical examples of integration, we aim to categorize these interventions into a resource healthcare practices can utilize to self-evaluate their practices and identify opportunities for collaboration with PH.

Given the strong interest across jurisdictions to address inequities in care, we further hope our findings can be utilized by healthcare practices to match health services to unique population health needs and thereby target underserved, high-risk populations groups.

## Methods

We conducted a scoping review using an updated version of Arksey and O’Malley’s [26] scoping study methodology developed by Levac et al. [27] A scoping review is a form of knowledge synthesis that utilizes narrative integration to systematically chart relevant evidence in research related to a broadly defined area and map the results according to key issues or concepts [26, 27].

### Search strategy

MEDLINE, EMBASE and PsycINFO electronic databases were searched from 1990 to 2017 for articles containing key words or MESH terms related to “Public Health”, “Primary care”, “Population health approach”, “Collaboration”, and “Integration”, along with all related terms. This was an iterative process and the search strategy was refined several times by combining different key words using the Boolean operators “AND” and “OR”. Our combined searches yielded 2375 articles (after duplicates were removed). After screening titles and abstracts for relevance to PC and PH collaboration or integration, 366 articles were retained.

### Study selection and inclusion criteria

We restricted our search to studies in English, published between 1990 and 2017, and published in a peer-reviewed journal with any of the following study designs: randomized controlled trials, cluster-randomized controlled trials, cluster-controlled studies, observational cohort studies, case reports and series, project evaluations, and review articles. Furthermore, studies were included in our scoping review if the reported collaboration was based in Canada, United States, Western Europe, Australia and New Zealand to allow comparability of health systems in terms of training, range of clinical governance and funding mechanisms [24]. Finally, similar to the approach by Martin-Misener et al. [24], we limited our review to studies that reported on structures, processes, outcomes or markers associated with PC-PH collaboration.

A web search was conducted to review the grey literature and we retrieved relevant reports published through websites of various associations and research networks. The reference lists of identified articles were reviewed for additional sources. Overall, the study selection criteria and reference lists yielded a total of 45 articles for final analysis.

### Data extraction and synthesis

Data were extracted from the final studies using a structured abstraction process (forms available on request from the corresponding author, M.S.). Standardized forms were created and entered into Excel by one author (M.S.) and data allocations were independently reviewed by two other authors (A.D.B., and R.U.) and verified for completeness. Discrepancies and methodological quality of selected studies were discussed and resolved through consultations among three authors (M.S., A.D.B., and R.U.).

Applying a narrative approach [27], extracted data included year, location and context of where collaboration occurred, research methods of authors/organization, objectives and purpose of collaboration, specific intervention leading to collaboration, and description of intervention (structures, processes, outcomes or markers of collaboration). Furthermore, we utilized a series of determinants for collaboration developed by Roz Lasker and the Committee on Medicine and Public Health [28] to guide this data extraction process and the categorization of studies included in our review. Specifically, the classification process involved an adapted version of Lasker’s models of Medicine and Public Health Collaborations, which the authors reported as ‘Synergies’. Lasker defines these synergies as combinations of resources and skills utilized by professionals in medicine and public health, along with other partners in the community, to allow effective cross-sectoral collaborations [28].

## Results

**Fig. 1:**
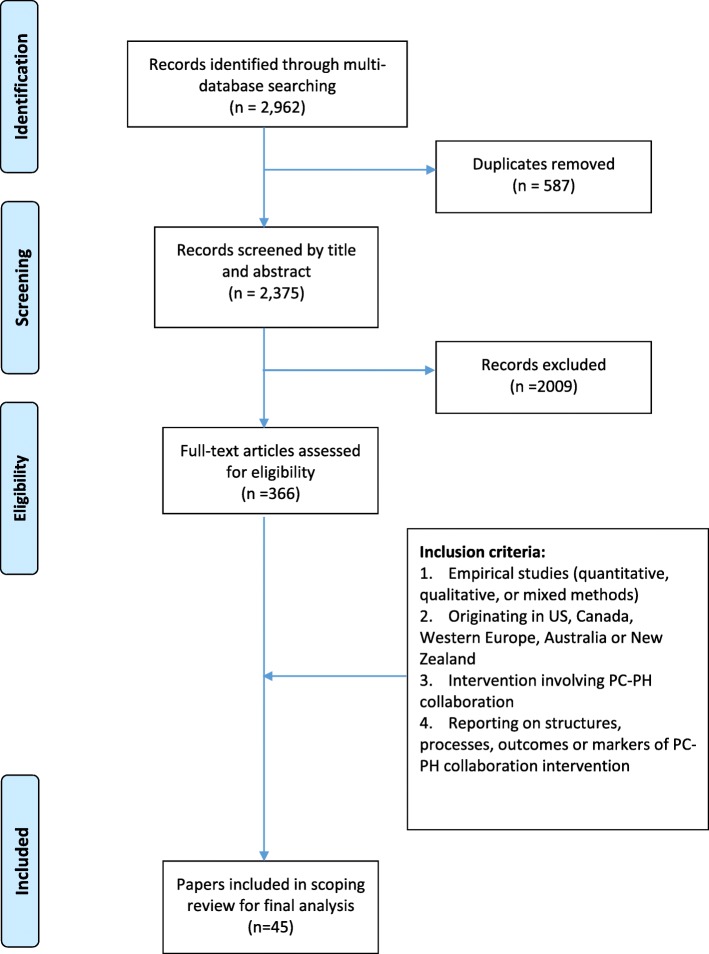
PRISMA flowchart of study selection process

Of the 2962 studies identified by the search strategy, 45 met our inclusion criteria and were selected for the final analysis, as illustrated in a PRISMA flowchart outlining the search and screening process involved in selection of articles for the scoping review (Fig. 1).

The final 45 studies that described interventions leading to PC-PH collaborations were classified based on an adapted version of Lasker’s models of Medicine and Public Health Collaborations, which the authors reported as ‘Synergies [28].

We focused on the following four synergies:

1. Coordinating healthcare services (Table 1);
2. Applying a population perspective to clinical practice (Table 2);
3. Identifying and addressing community health problems (Table 3);
4. Strengthening health promotion and health protection (Table 4).

Importantly, these combinations encompass all health professionals and organizations across both health sectors, and also involve various areas of PC and PH including clinical practice, health policy, education and research [28].

### Synergy 1: coordinating healthcare services

It has been well documented in the literature that a core strategy for promoting cross-sectoral collaboration between clinical care and PH is the coordination of healthcare services. Thirteen papers included an intervention leading to PC-PH collaboration that was categorized under Synergy 1.

Interventions and select empirical examples from the literature:

A. *Coordinate clinical services with community services* (*n* = 5) *–* clinical services, such as prevention, diagnosis and treatment/rehabilitation are combined with services including counselling, outreach and social programs [29–33]*.*
  1. Auerbach et al. - PH services for STDs, TB and HIV were combined with community health centers and Accountable Care Organizations as part of a CDC-STD collaborative to address service needs for high risk, stigmatized and uninsured sub-populations [29].
  2. Saeed et al. - Integrated health and social care commissioning as part of NHS Hounslow clinical commissioning group to develop prevention programs that ensure early diagnosis and improve outcomes, especially for long-term conditions [32].
B. *Bring other sector’s personnel to existing practice sites* (*n* = 6) *–* outside personnel are brought in to provide desired, individual-level support services to patients [31–37].
  1. Lebrun et al. –
    - Health centers held a long-term lease in the local health department; expanded through development of an internal referral system for services offered exclusively by each party [31];
    - Health centers hired community health workers to provide care coordination services for centres’ most complex and costly chronic care patients (services included assistance navigating healthcare system and addressing their social determinants of health [31].
  2. Hogg et al. - Short-term outreach intervention by PH nurses led to significant improvement in uptake of best practices for control of respiratory infections in PC centers [35].
C. *Establish “one-stop” centers organized around needs of local populations* (*n* = 7) *–* clinical and community-based professionals and programs are brought together at one site [29, 33, 37–41].
  1. Kempe et al. - Pediatric and family medicine services in Colorado were merged with a common PH department leading to significant increases in influenza immunization rates [37].
  2. Pickens et al. - Parkland Health System in Dallas TX has established a partnership with community organizations to provide other health and social services, creating a “one-stop” shopping net- work covering all PC disciplines and dental health [41].
  3. Adany et al. –
    - The Swiss-Hungarian Cooperation Programme developed a general practitioner (GP)-centered cluster model for community-oriented PC services;
    - Each GP cluster houses PH resources and health professionals including community nurses, dieticians, physiotherapists and health psychologists;
    - GP cluster practice sites address local population needs through new services including health promotion activities, health status assessments, lifestyle counselling, medical risk assessments, and chronic care rehabilitation services [38].

**Table 1:** Synergy 1 – Coordinating healthcare services

Intervention	Key features
A. Coordinate clinical services with community services (across different sites)	Combining clinical services (diagnosis, prevention, treatment rehabilitation) with:
	1. Counseling and educational services directed at personal risk behaviors, the management of particular health problems, the use of health services etc.
	2. Outreach and case management services to identify health needs of individuals and promote compliance with complex treatment programs
	3. Social services that address socioeconomic determinants of health
B. Bring other sector’s personnel (E.g. public health) to existing practice sites	1. PC sites can lease certain services from PH departments, and vice versa
	2. Organizations can hire or contract professionals with expertise or experience in providing a desired service
	3. PC or PH sites bring in outside personnel to provide individual-level support services for patients
C. Establish “one-stop” centers (geographic proximity)	1. Co-location of both sectors’ services to promote geographical proximity of PC and PH professionals and programs
	2. Addressing health inequalities through “one-stop” centers located in disadvantaged regions and organized around needs of local populations

### Synergy 2: applying a population perspective to clinical practice

While the previous synergy outlined how the two health sectors can strengthen each other through coordination of their respective services and programs, synergy 2 offers an approach of integrating the individual patient-level and population-level perspectives of PC and PH. Twenty-one papers included an intervention leading to PC-PH collaboration that was categorized under Synergy 2.

Interventions and select empirical examples from the literature:

A. *Use and share population-based information to enhance clinical decision-making* (*n* = 8) *–* information includes population-specific health problems and risks, underlying causes of health issues, and local health resource availability [13, 29, 31, 36, 42–45].
  1. Harris et al. - Sharing of information on immunization and respiratory disease management among US State health department, American Academy of Family Physicians, and American Academy of Pediatrics led to improvement in vaccination coverage levels and enhanced emergency immunization responses in event of pandemic influenza or natural disasters [42].
  2. Perry et al. - Healthcare collaborative in Trenton NJ across hospitals, Division of Health, and over forty community organizations used data sharing to develop a community health improvement plan and allocate community health resources to target unique health needs of the population [45].
B. *Use population-based strategies to “funnel” patients to medical care* (*n* = 9) – strategies include community-wide screening, case-finding, and outreach programs [40, 43, 46–52].
  1. Alexy et al. - The Rural Mobile Health unit (designed for elderly, at-risk populations in two rural counties in the US) offered various screening and outreach programs conducted by PC and PH staff that resulted in increased breast and cervical cancer screening rates, as well as improved immunization coverage for influenza, pneumonia and tetanus [50].
  2. Mayo et al. - The homeless shelter TB program in Charleston SC established CDC-recommended TB screening protocols for shelter guests; the mass-screening process was carried out by medical staff, PH nurses and university nursing students as part of a community collaboration that contributed to the reduction of TB infection and disease in the homeless shelter population [52].
  3. Heller et al. - Community-based mobile PC clinics in Maryland target underserved populations and provide community-wide screenings (colonoscopies, mammograms, breast exams, pap smears and pregnancy tests) through PC staff and referrals to local health centers, hospitals, health departments and community agencies; secondary referrals are made to specialist providers for patients with complex conditions requiring diagnostic testing, prescriptions, social services [48].
C. *Use population-based analytic tools to enhance practice management* (*n* = 7) – tools include risk assessment, cost-effectiveness analysis, and are used towards information about population health status, risks and service needs [17, 30, 31, 35, 44, 53, 54].
  1. Pickens et al. – As part of its evaluation strategy, the Parkland community-oriented primary care (COPC) model in Dallas TX:
    - assesses health outcomes and data on costs of healthcare services; evaluation and outcome studies such as community assessment surveys are used to measure access to PC services, service use among adults, infant mortality rates, length of inpatient stays, Medicaid coverage rates, charges incurred by patients etc.
    - conducts cost-effectiveness analyses using outcome studies, efficiency studies and information on changing demographic patterns;
    - program assesses community needs using information on population variables such as age, ethnicity, and income; birth and birth-related factors; death rate variables; access to primary care; and hospital use;
    - Data from the Parkland COPC community assessment tool helps allocate community health centers and PH outreach activities, and forms basis for health outcome measures and evaluation of community benefits [41].
  2. Hogg et al. – Primary care centers in Ottawa ON utilized outreach facilitation with public health professionals to improve respiratory infection control practices through audit of current practice, evidence-based best practices, planning and consensus building, and feedback on performance change [35].

**Table 2:** Synergy 2 – Applying a population perspective to clinical practice

Intervention	Key features
A. Use and share population-based information to enhance clinical decision-making	1. Population-based information includes data about prevalent health problems, health risks within the community, and preventive services for particular patient groups
	2. Collaboration can bring together governmental PH agencies, medical societies, and academic institutions to address topical issues of importance to all sectors (E.g. communicable diseases)
	3. Healthcare practices can utilize interventions involving population-based information to determine health resource availability in particular geographic regions
B. Use population-based strategies to “funnel” patients to medical care	1. Population-based strategies include community-wide screening, case finding, and outreach programs
	2. Collaboration partners can strengthen the traditional “screen and treat” strategy in two ways: by improving the effectiveness of the screening process itself, and by assuring that all individuals identified as having problems on screening tests receive appropriate follow-up care
C. Use population-based analytic tools to enhance practice management	1. Analytic tools include clinical epidemiology, risk assessment, cost-effectiveness analysis, and performance measurement/evaluation
	2. These analytic tools can support medical sector organizational planning through use of information about population health status, risks and service needs (therefore can inform decisions about practice site locations, service provision at each site, practice staffing patterns, need for patient education programs etc.)

### Synergy 3: identifying and addressing community health problems

In contrast to the previous synergy where interventions involved applying a PH lens and population perspective to medical practice, synergy 3 switches the focus and targets opportunities present in clinical care in order to advance broader community-based goals of PH. Nineteen papers included an intervention leading to PC-PH collaboration that was categorized under Synergy 3.

Interventions and select empirical examples from the literature:

A. *Conduct community health assessments* (*n* = 8) *–* information collected can be helpful in identifying and prioritizing community health problems [17, 29, 31, 41, 45, 55–57].
  1. Perry et al. - A PC-PH partnership in Trenton NJ implemented a unified community needs assessment (after previous attempts with separate assessments) and engaged 40+ community organizations; the partnership centers identified specific health needs voiced by community members and allocated local healthcare resources accordingly [45].
  2. Pickens et al. - The Parkland community-oriented PC program in Dallas TX assesses community needs and health outcomes through outcome studies (E.g. community assessment surveys), efficiency studies, information on changing demographic patterns, a range of information on population variables including age, ethnicity and income, birth and birth-related factors, death rate variables, access to PC, and hospital use [41].
B. *Use clinical encounters and share data to build community-wide databases* (*n* = 10) *–* standardized systems, e.g. Electronic health records (EHRs), used to collect and disseminate data. Collaborations can utilize variety of systems including EHRs, vital records, communicable disease surveillance systems etc. [14, 31, 43, 44, 46, 58–62].
  1. Calman et al. - Through a series of collaborative EHR data sharing initiatives in 2003, a partnership between the New York City Department of Health (NYCDOH) and Institute for Family Health (IFH) addressed the mutual PH and PC goals of advancing surveillance and clinical management of communicable and chronic diseases:
    - Syndromic surveillance practices were improved through automated EHR data reporting to PH departments in order to monitor patient encounters for symptoms associated with particular infectious diseases;
    - Disease monitoring through registries and physician reporting was enhanced by incorporating alerts within the shared EHR system to notify physicians of reportable diagnoses and conditions at the point of care;
    - EHR alert system uses epidemiological findings from PH investigations to inform PC providers of various conditions, such as when the NYCDOH notified IFH and affiliated PC practices about detection of West Nile Virus (this led to EHR-based prompts reminding physicians to look for patients with particular symptoms E.g. fever and headache);
    - EHR data helped PC practices identify patients at particular risk of developing certain chronic conditions; as a result IFH promoted patient knowledge/awareness around disease risk factors and encouraged preventive behavior (vaccinations, cancer screenings diabetes awareness etc.) and patients were guided towards appropriate PH resources (E.g. mammography screening programs);
    - EHR data, such as patient demographics, helped address racial and ethnic disparities in healthcare. E.g. by including patients’ race, ethnicity and language in the EHR system, IFH identified disparities in Hemoglobin A1c levels of diabetic patients; this led to creation of the Enhanced Diabetes Care Model that provides further outreach and care management for patient groups who are underserved/experiencing barriers to care [43].
C. *Use clinical opportunities to identify and address underlying causes of health problems* (*n* = 4) *–* important health associations to social, physical, environmental determinants can be made and followed up with targeted education or counselling for risk factors and personal behaviours [14, 30, 32, 57].
  1. Gosling et al. –
    - As part of the “Healthy Liverpool Program” in the UK, physicians worked with PH and local communities to identify underlying causes of ill health; authors noted that collaborative interventions targeted and addressed a variety of disease risk factors including blood pressure, smoking status and BMI of patients;
    - PC teams partnered with environmental health and housing as part of the Healthy Homes Initiative to coordinate efforts to provide at-risk patient groups with adequate and safe housing options [14].
  2. Bourdages et al. – An intersectoral community initiative in Quebec implemented an integrated prevention program for cardiovascular disease and lung cancer by promoting health behaviours and reducing modifiable risk factors; health promotion interventions were specific to local communities, focused on improving patient knowledge and healthy behaviours, and ranged from promoting physical activity to reduction in tobacco use [54].

**Table 3:** Synergy 3 – Identifying and addressing community health problems

Intervention	Key features
A. Conduct community health assessments	1. Facilitates planning and development of health programs and services (for both PC and PH):
	2. Ensures that health programs and services offered by PC and PH are responsive to local community needs
	3. Allows efficient allocation of limited health resources
	4. Community health assessments are more robust if they aggregate data from multiple sources: quantitative data from EHRs, administrative databases or surveys, and qualitative information from community meetings, interviews, and focus groups (and if the data is analyzed from multiple perspectives)
B. Use clinical encounters and share data to build community-wide databases	1. Collaborations can draw on a broad range of data systems including electronic health records, vital records such as electronic birth certificates, surveillance systems targeted at communicable diseases, antibiotic resistance, or behavioral risk factors, and disease-specific registries centered around as cancer, trauma, asthma, tuberculosis, immunization etc.
	2. Input from public health practitioners is valuable in elucidating how the information in the system would be used to monitor health outcomes, to determine where clinical services are being delivered, or to target outreach efforts and media campaigns
	3. Public health data can be utilized to produce reports on health and disease status of patients at the primary care practice level. This helps to understand needs of practice population and identify specific actions to address local health needs
	4. Practice lists can be utilized to design health interventions, track health outcomes, and target specific high-risk patient populations
	5. Standardized demographic data in health care settings that can identify gaps and point toward best practices for eliminating disparities
C. Use clinical opportunities to identify and address underlying causes of health problems	1. Collaborations focus on health problems with prominent environmental, social and behavioral risk factors such as lead toxicity, tobacco use, and domestic violence
	2. Patients are be provided with targeted counselling and educational materials about personal behaviors such as smoking, sedentary lifestyle or heavy drinking, and referred to appropriate community programs
	3. Connections can be made to patients’ social or physical environment in order to test a disease contact or assess potentially toxic worksites/homes

### Synergy 4: strengthening health promotion and health protection

Until now we have explored the roles of PC and PH in strengthening each other’s respective functions through coordinating healthcare services, applying a population lens to clinical care, and utilizing clinical opportunities to address health problems at the community level. The final synergy takes a broader view of the population-based approach to healthcare delivery and outlines interventions to strengthen health promotion and health protection. Twenty-one papers included an intervention leading to PC-PH collaboration that was categorized under Synergy 4.

Interventions and select empirical examples:

A. *Health promotion through education* (*n* = 11) *–* counselling or educational materials can be organized around the prevention and management of a particular disease, or can target personal health behaviours [32, 34, 38, 39, 44, 50, 60, 63–66].
  1. Alexy et al. - PC and PH staff working in a Rural Mobile Health Unit delivered educational seminars/materials on influenza vaccinations, indications and benefits of breast cancer screening for elderly women, and healthy sleep hygiene practices; efforts led to improved rates of breast cancer screening and immunization, and an increase in patient knowledge of PC services available in the local US counties [50].
  2. Adany et al. - Community-based GP groups and PH professionals in Hungary offered services including initial health status assessments followed by referrals to community health promotion programs for patients presenting without clear health risks; individuals with identifiable behavioral risks were referred to specific lifestyle counselling services to provide education around personal risk behaviors [38].
B. *Advocate for health-related laws/regulations, and for disadvantaged groups* (*n* = 4) *–* advocating by drawing on scientific expertise of both health sectors, as well as the influence they have with general public and policymakers [13, 14, 55, 67].
  1. Kuo et al. - Recognizing that the traditional medical approach in PC and pediatrics is limited in addressing underlying social and environmental determinants of children’s health, authors of a study out of the UCLA Center for Healthier Children, Families and Communities highlight several roles PC and PH professionals can play in advocating for healthy public policy:
    - In the area of asthma control, PC/PH professionals can advocate for more stringent enforcements of regulations on hazardous air and environmental pollutants;
    - PC pediatricians can conduct environmental health assessments as part of their standard care practices for patients with asthma, obesity or repeated injuries (assesses for presence of structural hazards in the home that can contribute to development of asthma and lead poisoning);
    - To tackle childhood obesity, healthcare professionals can advocate for community-based initiatives to promote walkability and cycling, and increasing availability of fresh fruits and healthier food choices in stores close to school areas;
    - PC/PH professionals can inform patients/families about community resources for mental health, and should also advocate the need for comprehensive care models for mental health and substance use in children and adolescents;
    - PC pediatricians can also advocate reducing children’s exposure to violence and collaborate in policy/legislative circles to promote public awareness around impact of violence exposure on children’s development [67].
C. *Launch “Healthy Communities” Initiatives* (*n* = 7) *–* community-wide projects that bring the public, private, and non-profit sectors together to develop solutions to community health problems [14, 17, 28, 41, 45, 68, 69].
  1. Gosling et al. – PC-PH Initiatives can be developed on a community-wide scale, as demonstrated by the “Healthy Liverpool Program” in the UK which implemented a series of projects to promote city-wide population health:
    - “Smoke-Free Liverpool” is a project that takes a multi-pronged approach by advocating for local smoke-free legislation, encouraging voluntary smoke-free workplace policies, and providing comprehensive smoking cessation services to city residents;
    - Projects also addressed underlying causes of health problems. E.g. PC practices in identified low Vitamin D levels in a Somali population and designed health promotion activities and screen-and-treat programs targeted at these groups;
    - Projects such as the “Healthy Homes Initiative” tackle broader determinants of community health; this initiative involves collaboration between PC, environmental health, and housing services to mobilize community resources and provide at-risk populations with safe and affordable housing options;
    - Low-income patient groups facing financial hardship can be referred to the Citizens Advice Bureau by GPs as part of the “Advice on Prescription” project to offer individuals with advice on welfare, benefits, debt and housing [14].
  2. Pickens et al. –
    - Parkland Health System’s Community-Oriented PC (COPC) model in Dallas TX is organized around six key elements: [1] assessment of community needs/assets, [2] community prioritization of healthcare issues, [3] collaboration with community groups, [4] provision of primary healthcare, [5] evaluation, and [6] financing;
    - Particularly noteworthy is their development of the Community Health Improvement Measurement and Evaluation System (CHIMES) to document areas where community health investment has produced savings (E.g. fewer hospitalizations, fewer days lost from work, less school absenteeism etc.) [41].

**Table 4:** Synergy 4 – Strengthening health promotion and health protection

Intervention	Intervention features and empirical examples
A. Health promotion through education	1. Counselling and educational materials can be targeted at prevention and management of particular conditions such as infectious diseases, chronic diseases, and cancer:
	2. Integration efforts can concentrate on personal risk behaviors related to health, including cigarette smoking, substance abuse, risky sexual activity, poor diet, and physical inactivity:
	3. This intervention provides opportunities for the PC sector to contribute to health promotion and education campaigns led by PH and local health authorities:
	4. Information about environmental issues, such as hazardous wastes, lead poisoning, and fluoridation, is provided in some materials; other materials can provide patients with a list of available health resources.
B. Advocate for health related laws/regulations, and for disadvantaged groups	1. Collaboration efforts target a broad range of community issues such as alcohol and tobacco control, vehicular injury, water fluoridation, cycling and walking infrastructure, gun control, as well as a number of health equity issues including income adequacy, affordable housing, and early childhood education supports etc.
	2. PC and PH professionals involved in these forms of public policy advocacy can draw upon various non-clinical tools/resources such as scientific/technical expertise, lobbying and public relations skills, and influence with policymakers and the public in order to influence regulations that promote conditions more conducive to safety and well-being of populations
C. Launch “Healthy Communities” Initiatives	1. Initiatives can be targeted at particular health problems or needs specific to groups within the community
	2. PC-PH Initiatives can also be developed on a larger, community-wide scale through multiple projects designed to promote health of local populations
	3. These projects go beyond categorical health promotion activities by developing a broad-based process to tackle multiple community health issues, and evaluative mechanisms to determine outcomes and benefits of institutional investments in community health status improvement

Two additional areas for collaboration noted in Lasker’s report are ‘improving access to care for the uninsured’ and ‘collaborating around policy, training, and research [28]. As highlighted through Lasker’s models of Medicine and Public Health Collaborations, the former can be implemented through various interventions such as establishing free clinics and referral networks, increasing clinical staffing at public health facilities, and transferring uninsured patients to mainstream medical settings. The latter model can be achieved through notable interventions that include engaging in cross-sectoral education and training, as well as conducting cross-sectoral research. Although the studies in our review did not address these areas consistently enough to warrant their inclusion under separate synergies within our findings, these interventions can be of value for practices developing integration initiatives.

## Discussion

### Strengthening respective functions of clinical care and public health sectors through collaboration

Each of the examples of interventions identified in the literature feature a conceptually novel aspect of healthcare delivery – cross-sectoral collaboration through coordination of a range of services traditionally provided independently of each other. By linking clinical services to those of other sectors such as PH, as highlighted in synergy 1 (*coordinating healthcare services*), healthcare practices can enhance patient follow-up and improve health outcomes [11], reduce duplication of services, and achieve economies of scale by centralizing services across PC and PH sites [11, 28, 30].

Several interventions found in our study largely focus on the clinical end of the PC-PH collaboration spectrum. However, as collection and analysis of population-based information are core functions of PH, it is also important to touch upon the benefits to this sector. Synergy 2 (*applying a population perspective to clinical practice*) offers strategies that can be useful and cost-effective for PH agencies collaborating with PC. For example, integration can facilitate translation of epidemiologic findings developed from population health tools into the clinical practice setting. Additionally, these integration efforts can ensure that patients identified by PH screening programs receive appropriate referral and follow-up services [48, 49]. Furthermore, integration efforts under Synergy 3 (*identifying and addressing community health problems*) also target opportunities present in clinical care to advance broader community-based goals of PH.

Significantly, as shown consistently across several interventions and most notably under intervention 3B (*using clinical encounters and sharing data to build community-wide databases)*, collaboration between PC and PH sectors has been greatly enhanced through recent advancements in health information technology (IT) [14, 31, 43, 44, 46, 58–62] and the growth of population health informatics [70]. Widespread adoption of EHR systems across hospitals in countries such as the US, along with greater involvement of hospitals and academic health centres in health IT exchange initiatives has facilitated community needs assessments, design of population-level health interventions, and tracking of health outcomes in specific patient populations [43, 44, 71].

Another important finding is that interventions across our review findings are not mutually exclusive and often overlap in the empirical studies identified. In particular, interventions under Synergy 4 (*strengthening health promotion and health protection*) often incorporate strategies from other models of collaboration which, importantly, highlights the mutually reinforcing nature of the synergies and the value in employing a range of interventions to achieve integration. Cross-sectoral efforts targeting health promotion and protection are becoming increasingly important in the current healthcare environment with rising burden of illness and disability due to health problems involving violence, substance abuse, chronic disease and environmental hazards, among others [3, 28]. Efforts at addressing such health problems associated with a variety of social, behavioral and environmental risk factors will have to go beyond the traditional biomedical model and employ a population-based approach to promote the health of communities.

### Addressing inequities in care

From a health equity perspective, our review findings also provide several empirical examples of interventions that address inequities in care by targeting underserved, high-risk populations groups.

Under intervention 1C (*establishing “one-stop” centers*), for example, the Swiss-Hungarian Cooperation Programme developed a general practitioner-centered cluster model for community-oriented PC services in the most disadvantaged regions of Hungary to provide low-income patients access to various new services such as health status and risk assessments, lifestyle counselling, and chronic care rehabilitation [38]. Additionally, within intervention 2B (*using population-based strategies to “funnel” patients to medical care*), Heller et al. describe a community-based mobile PC clinic that aims to increase access to healthcare services for underserved populations in Maryland through community-wide screening and secondary referrals to specialists for patients with complex conditions [48]. Also under the same intervention, we found initiatives targeting TB patients in homeless shelters through implementation of CDC-recommended mass-screening protocols [52], as well as Rural Mobile Health Units designed for elderly, at-risk populations with limited access to PC services [50].

Furthermore, under intervention 3B (*using clinical encounters and sharing data to build community-wide databases*), one collaboration effort utilized shared EHR data, such as patient demographics, to identify disparities in Hemoglobin A1c levels of diabetic patients which resulted in creation of the Enhanced Diabetes Care Model for certain racial and ethnic groups experiencing barriers to care [43]. A notable example of addressing inequities in care within intervention 4C (*launching “Healthy Communities” initiatives*) is provided by Gosling et al. where authors highlight an effort by PC practices in Liverpool, UK to identify low Vitamin D levels in a Somali population and design health promotion activities and screen-and-treat programs targeted at these groups [14]. As part of this same city-wide project, and with a focus on the broader determinants of community health, the “Healthy Homes Initiative” involves collaboration between PC, environmental health, and housing services to mobilize community resources and provide at-risk populations with safe and affordable housing options. Lastly, in an example of a collaborative community-oriented PC model in Dallas TX, authors reported significantly better neonatal mortality outcomes, than those for the US overall, for African American and Hispanic populations residing in Texas [41].

### Implications for policy and practice: a population-based approach to address community health needs

Previous reviews on PC and PH collaboration have highlighted various aspects of developing cross-sectoral partnerships such as core principles for effective integration, facilitators and barriers, and overlapping functions and activities of both domains [11, 23–25]. However, these reviews have not found any significant efforts to produce practical tools for healthcare practices to enable implementation of collaboration. Our scoping review identified a range of interventions designed by organizations in health systems around the world that facilitated cross-sectoral integration. We subsequently categorized these interventions into Lasker’s synergy groupings which are combinations of resources and skills required to achieve collaboration [28].

Importantly, our review findings have been organized into a self-evaluation tool that can serve as a resource for identifying opportunities for cross-sectoral collaboration and allow practices to focus on pressing health needs facing their communities. More specifically, to facilitate the brainstorming process, clinicians and PH practitioners can utilize the tool to address questions such as the following:

1. *What services are currently being provided by our practice?*
2. *What are the current health and service needs in our community and which of these listed interventions address our identified gaps?*
3. *Which of these interventions are useful and feasible for adoption in our practice and what are the necessary steps required to pursue collaboration through these identified interventions?*

This tool can be found at the end of the article in Additional file 1: Self-evaluation tool for clinical practices to identify opportunities for collaboration with public health. The scoring systems within this tool have been adapted from *‘Self-assessment tool for the evaluation of essential public health operations in the WHO European Region 2015’* [72] and can be useful for practices in determining the level of progress made with respect to each intervention as well as identifying key areas for improvement for achieving collaboration.

One of the benefits of the population health approach is identifying community health needs and reorienting health care delivery and services to these needs [11, 17, 38]. Given the strong interest across jurisdictions to match health services to population health needs, the tool can be utilized by healthcare practices and organizations as part of their criteria for selecting community-based projects to move collaboration forward with. Further, by using the list of interventions outlined in our findings, both sectors can also come together to design interventions tailored towards underserved, high-risk population groups.

In providing healthcare organizations with a range of interventions implemented across different jurisdictions, our tool can be utilized to initiate practice-level discussions around implementation of integration efforts that are useful and feasible for all sectors involved. We hope this self-evaluation process will facilitate development of practical action steps required for practices to operationalize collaboration towards a population-based approach to integrated healthcare delivery.

## Limitations

Firstly, very few studies applied rigorous evaluation methods or reported on measures of successful collaboration. There is considerable difficulty in establishing acceptable outcomes and investments in community health are often only realized in the long term in other social and economic sectors [21, 41]. Further, the outcome measures that were reported by authors were often not robust. This may in part be due to the difficulty in specifying and measuring community care outcomes, and also because most collaborations noted in the literature were in the early stages of their development, making available outcomes limited. There is also inconclusive evidence to suggest which of the identified interventions are particularly exemplary in terms of impact or ease of implementation. Additionally, the interventions outlined in our findings vary in terms of the resources and activities needed for collaboration. For these reasons, we are limited in our ability to compare the effectiveness of adopted interventions and assess which programs are sustainable in the long term.

It is also important to note that the role of the PH sector in our findings is limited in terms of its scope and definition. The listed interventions tend to highlight the clinical aspects of PH and their functions. In doing so, they reveal certain gaps within the current literature on PC-PH collaboration. Examples of additional areas within PH that should also be considered include collaboration with local municipalities and addressing the other social determinants of health such as education. Importantly, the US IOM report on integrating PC and PH highlights that there is a growing evidence lending support to the value of undertaking such activities to address the full scope of PH functions. It is worth mentioning, though, that Synergy 4 (*strengthening health promotion and health protection*) does take a step towards bridging this gap by incorporating a wider range of PH functions such as health promotion initiatives through a focus on education, as well as creation of community-wide projects to involve public, private and non-profit sectors in developing solutions to community health issues.

## Conclusions

Our scoping review maps a series of specific interventions implemented by healthcare organizations internationally that facilitated collaboration between clinical care and PH. By analyzing empirical examples of all the interventions that led to integration, we have developed a self-evaluation tool to provide clinical practices with a resource to identify opportunities for collaboration with PH that address unmet health and service needs in their local communities.

Previous reviews on PC and PH collaboration have highlighted various aspects of developing cross-sectoral partnerships such as core principles for effective integration, facilitators and barriers, and overlapping functions and activities of both domains. We recommend that healthcare organizations use our tool in conjunction with these high-level principles noted in earlier reviews to help guide the design and execution of collaboration efforts geared towards a population health approach.

Further research in this area should be directed at developing specific outcome measures to compare effectiveness of collaboration interventions that lead to community health status improvement. This will provide additional guidance for practices and organizations in determining which of the currently identified interventions are likely to be feasible to implement and achieve sustainable health outcomes for local populations in the long term.

## Additional file

Additional file 1: Self-evaluation tool for clinical practices to identify opportunities for collaboration with public health. (XLSX 73 kb)

## Data Availability

The datasets analysed during the scoping review study (i.e., the raw coded data extracted from identified papers) are available from the corresponding author on request.

## Abbreviations

BMI: Body mass index
CCGs: Clinical commissioning groups
CDC: Centers for disease control
COPC: Community-oriented primary care
EHR: Electronic health record
GP: General practitioner
IOM: Institute of medicine
NHS: National health service
PC: Primary care
PH: Public health
STDs: Sexually transmitted diseases
TB: Tuberculosis
UCLA: University of California Los Angeles
WHO: World Health Organization
